# An unexpected cause of abdominal pain or maybe more

**DOI:** 10.11604/pamj.2019.32.102.18015

**Published:** 2019-03-04

**Authors:** Petros Ioannou

**Affiliations:** 1 Internal Medicine Department, University Hospital of Heraklion, Stavrakia & Voutes Crossroad, Heraklion, Crete, Greece

**Keywords:** Dissection, splenic infarct, thrombosis, abdominal pain

## Image in medicine

A 61-year old Caucasian woman, presented to the Emergency Department due to a four-week history of vomiting, diarrhea and abdominal pain. Physical examination revealed tenderness in the upper abdomen and normal bowel sounds. A CT was performed and the patient was admitted. A CT scan of the chest and the abdomen revealed a chronically dissected Stanford B thoracoabdominal aneurysm with mural thrombus, thrombosis of the celiac, hepatic, splenic and superior mesenteric arteries with collateral circulation, thrombosis of the portal and superior mesenteric veins and a splenic infarct. Dissection of an aneurysm is a catastrophic complication with high mortality and morbidity, thus an invasive treatment is often warranted with either surgery or endovascular repair. On the other hand, portal thrombosis is associated with inherited or acquired procoagulant states (like Factor V Leiden), hematologic diseases (like myeloproliferative diseases), cirrhosis, abdominal surgery or trauma and intra-abdominal inflammatory conditions. Its treatment involves anticoagulation or rarely, thrombolysis or thrombectomy. Both chronic dissection of the abdominal aneurysm and the portal vein thrombosis can cause abdominal pain, nausea, and other gastrointestinal symptoms secondary to bowel hypoperfusion. Diagnosis: chronically dissected Stanford B thoracoabdominal aneurysm and thrombosis of the celiac, hepatic, splenic and superior mesenteric arteries with collateral circulation, thrombosis of the portal and superior mesenteric veins and a splenic infarct.

**Figure 1:**
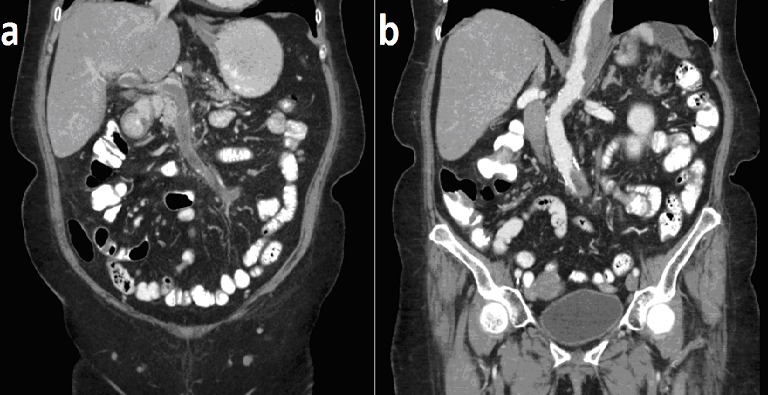
(A) shown are images of the CT scan of the patient with oral contrast medium, without and; (B) with intravenous contrast medium, showing a chronically dissected Stanford B thoracoabdominal aneurysm with a mural thrombus, thrombosis of the portal and superior mesenteric veins and a splenic infarct

